# Tobacco and the Worker

**Published:** 1922-03

**Authors:** 


### TOBACCO AND THE WORKER.

"AN Output Study of Users and Non-users of Tobacco in a Strenuous Physical Occupation" is the title of an article in the "Journal of Industrial Hygiene," by three members of the Laboratory of Physiology, Stanford University. In conclusion, the writers state that—

"From the foregoing data it appears that workers who chew have a much lower output-rate than those who only smoke, or who do not use tobacco in any form. The light smokers, however, do show some inferiority in output-rate, and the heavy smokers a very slight superiority, although these differences are too small to be statistically dependable. The difference between the light and heavy smokers is apparently significant. The fact that light smokers have a lower output-rate than heavy smokers is difficult to explain, but may be an indication that insufficient use of tobacco has more deleterious effects than a larger use, which might confer an immunity that would be lacking in the case of light smokers. This explanation is entirely a surmise, but should be followed up.

"The low output of chewers may be due to a greater absorption of nicotine into the system than takes place from smoking. Some reason for this is obvious when we consider that in smoking at least half the nicotine is lost in the smoke from the burning-point and that a large part of the nicotine in the inhaled smoke may be exhaled before it is absorbed. This is quite likely, as smoke can be drawn through several wash-bottles without losing all its nicotine, because the alkaloid is probably absorbed on the surface of liquid particles in the smoke which are notably difficult to absorb in a wash-bottle. In chewing tobacco, on the other hand, the saliva of the chewer seems to have ample opportunity to absorb the nicotine of tobacco, and from the saliva the mucous lining of the mouth may absorb the poison. Furthermore, in most chewing a certain amount of saliva is swallowed, which gives abundant opportunity for absorption of nicotine. It is possible, therefore, to offer an explanation for the fact that chewers have a lower output-rate than smokers on the basis of the relative nicotine-absorption, although until actual absorptive studies of the two groups have been made such an explanation can be only tentative.

"Conclusions : Smoking has little effect on output-rate in the strenuous physical occupation studied by us. (2) Chewing markedly lowers output-rate in this strenuous physical occupation. (3) Light smokers have a slightly lower output-rate than heavy smokers in this strenuous physical occupation."

We are inclined to wonder, says the "New Zealand Journal of Health and Hospitals," whether the use of tobacco had really any bearing upon the output of work in the above instances. For instance, we note that the largest output of work was from heavy smokers, these beating the non-smokers. Might not this be due to the fact that the man of robust constitution did not need to be a non-smoker, or even a light smoker, whereas his more delicate fellow might have had either to discontinue smoking altogether or only indulge it in great moderation ?

In the case of food faddists and faddists of all descriptions we are constantly faced with evidence supporting such line of reasoning. Entering a vegetarian restaurant and studying generally the diners therein, one sees an unhealthy-looking lot, as a rule, but it does not follow that their health is the result of their diet—the diet may be a result of their health. On the other hand, enter a rough dining-house where huge quantities of meat are consumed, and notice the strong, robust-looking crowd of diners therein. It can similarly be argued that their physical condition is not necessarily the result of their diet, but permits their diet, though possibly they are laying up for themselves vascular or renal troubles just in the same way as their fellows in the vegetarian restaurant may be aggravating rather than relieving dyspeptic troubles by swallowing stodgy masses of flatulent food.

Having all this in view, we are inclined to think that the amount of rock heaved by a healthy navvy is very little influenced by his diet, his drink, or tobacco, though doubtless if he ate, drank, and smoked in moderation and not in excess his general health would even be better, and the output consequently greater.

### HOSPITAL ACCOUNTS.

### How to Enter Patients' Payments.

As a matter of urgency, the three great Funds lately issued a preliminary circular, containing amendments to the form of Income and Expenditure account, showing how patients' payments and similar receipts on account of services to patients should be entered.

The following amendments to the headings and sub-headings on the Income side of the Income and Expenditure Account will apply to the accounts for the year 1921 and until further notice :—

In place of the present Income heading, "XI. Patients' Payments," and "XII., Receipts under the National Insurance Act," the following new heading and sub-headings are to be substituted :—

XI.—Receipts on Account of Services to Patients.

(*a*) From in-patients, (*b*) from out-patients, (*c*) from public authorities, (*d*) from approved societies, (*e*) from other sources

The heading "Other Receipts" will now be numbered XII. instead of XIII.

*Patients' Payments.*—The definition of these given in par. 19, page 8, of the Revised Uniform System, will apply to the new heading "Receipts on account of services to patients," whether from patients themselves, or from their friends, or from charitable agencies, or from public authorities, or from other sources on their behalf.

Under this paragraph, only "exceptional" voluntary payments, outside the rules of the hospital as to patients' payments, may be entered under the heading "Donations."

All contributions from or on behalf of patients resulting from applications made to patients to contribute towards the cost of services rendered should be classified under the heading XI. (*see above*).

*Receipts under the National Insurance Act.*—The definition in par. 21, page 9, of the Revised Uniform System will remain in force, but sums received will be entered under the new heading XI. (*a*) "from approved societies" if thence received, and under XI. (*c*) if received from a public authority.

The second part of par. 19, page 8, is cancelled

